# Integrative analysis of SF-1 transcription factor dosage impact on genome-wide binding and gene expression regulation

**DOI:** 10.1093/nar/gkt658

**Published:** 2013-08-01

**Authors:** Mabrouka Doghman, Bonald C. Figueiredo, Marco Volante, Mauro Papotti, Enzo Lalli

**Affiliations:** ^1^Institut de Pharmacologie Moléculaire et Cellulaire CNRS, Valbonne 06560, France, ^2^Associated International Laboratory (LIA) NEOGENEX CNRS, Valbonne 06560, France, ^3^University of Nice-Sophia-Antipolis, Valbonne 06560, France, ^4^Federal University of Paraná, Curitiba, Paraná 80060-000, Brazil, ^5^Instituto de Pesquisa Pelé Pequeno Principe, Curitiba, Paraná 80250-060, Brazil and ^6^Department of Oncology, University of Turin, Orbassano 10043, Italy

## Abstract

Steroidogenic Factor-1 (SF-1) is a nuclear receptor that has a pivotal role in the development of adrenal glands and gonads and in the control of steroid hormone production, being also implicated in the pathogenesis of adrenocortical tumors. We have analyzed the mechanisms how SF-1 controls gene expression in adrenocortical cells and showed that it regulates different categories of genes according to its dosage. Significant correlations exist between the localization of SF-1-binding sites in chromatin under different dosage conditions and dosage-dependent regulation of gene expression. Our study revealed unexpected functional interactions between SF-1 and Neuron-Restrictive Silencer Factor/RE1-Silencing Transcription Factor (NRSF/REST), which was first characterized as a repressor of neuronal gene expression in non-neuronal tissues, in the regulation of gene expression in steroidogenic cells. When overexpressed, SF-1 reshapes the repertoire of NRSF/REST—regulated genes, relieving repression of key steroidogenic genes. These data show that NRSF/REST has a novel function in regulating gene expression in steroidogenic cells and suggest that it may have a broad role in regulating tissue-specific gene expression programs.

## INTRODUCTION

Steroidogenic Factor-1 (SF-1/Ad4BP) is a transcription factor belonging to the nuclear receptor superfamily (NR5A1 in the nuclear receptor nomenclature) that was first identified as a regulator of the expression of steroidogenic P-450 enzymes in the adrenal cortex (1,2) and functions as a global regulator of steroidogenic gene expression [reviewed in (3)]. Furthermore, SF-1 has a pivotal role in adrenogonadal development and differentiation into the steroidogenic lineage: mice lacking *Sf1* have no adrenal glands and gonads, while its overexpression in the embryo induces formation of ectopic adrenal tissue (4–6). Multiple factors regulate SF-1 activity: association with positive and negative cofactors, post-translational modifications, phospholipid ligand availability, epigenetic regulations and gene dosage [reviewed in (7)]. In particular, alterations of SF-1 dosage, both in defect and in excess, produce relevant pathological effects. *Sf-1* +/− mice have hypoplastic adrenals and gonads (8) and impaired compensatory adrenal growth following unilateral adrenalectomy (9). In humans, heterozygote *NR5A1* mutations are mostly associated with disorders of sex development (10), with only a few cases also displaying adrenal insufficiency (11). On the other side of the spectrum, SF-1 overexpression increases adrenocortical cancer cell proliferation and induces adrenocortical tumor formation in mice (12) and in humans is associated to adrenocortical tumorigenesis both in children (13) and adults (14).

The aim of this work was to study how differences in SF-1 dosage are connected to regulation of gene expression in adrenocortical cancer cells and to investigate the mechanisms regulating SF-1 transcriptional activity by comparing its genome-wide chromatin-binding sites in conditions of basal and increased expression. Our results allowed us to unexpectedly identify functional interactions between SF-1 and Neuron-Restrictive Silencer Factor/RE1-Silencing Transcription Factor (NRSF/REST), originally described as a transcriptional repressor of neuronal differentiation genes in non-neural cells (15,16), in the regulation of gene expression in adrenocortical cells.

## MATERIALS AND METHODS

### Cell culture

H295R/TR SF-1 cells (12) were cultured in Dulbecco’s modified Eagle’s medium/F12 supplemented with penicillin/streptomycin, 2% NuSerum (BD Biosciences), 1% ITS+ (BD Biosciences), blasticidin (5 µg/ml, Cayla InvivoGen) and zeocin (100 µg/ml, Cayla InvivoGen). As a method of authentication, the cell karyotype and steroid secretion profile were periodically tested. To induce SF-1 overexpression, cells were treated with doxycyline (1 µg/ml, Sigma-Aldrich) for 3 days before being processed for chromatin immunoprecipitation (ChIP). HeLa cells were cultured in Dulbecco’s modified Eagle’s medium (4.5 g/l glucose; Invitrogen) supplemented with penicillin-streptomycin and 10% fetal calf serum (Invitrogen).

### Gene knockdown and expression profiling

For knockdown experiments, H295R/TR SF-1 cells were transfected with SF-1–specific (AGA GCC AGA GCU GCA AGA UCG ACA A) (12), NRSF/REST–specific (UAU CUU AAC AGG UUC CUU CUG GAC C) and control (medium GC) Stealth siRNA (small interfering RNA) oligos (Invitrogen) using the Amaxa nucleofection technique (Lonza). Cells were electroporated with 80 pmol siRNA/10^6^ cells using solution R and the T-020 program and then plated in 24-well plates. Total RNA was extracted 72 h after nucleofection by the RNeasy Mini kit (Qiagen). For SF-1 overexpression experiments, H295R/TR SF-1 cells were cultured in the presence or in the absence of doxycycline for 3 days in 24-well plates before RNA extraction. Total RNA was used for gene expression profiling on GeneChip HG-U133 Plus 2.0 (SF-1 overexpression experiments) or Human Gene 1.0 ST (SF-1 and NRSF/REST knockdown experiments) arrays (Affymetrix) using the manufacturer’s protocols. Three biological replicates were analyzed for both the knockdown and the overexpression experiments. Data were analyzed using the Affymetrix Command Console and Expression Console softwares. Transcripts were considered as differentially expressed when there was a 2-fold or greater difference in expression levels, with a *P* < 0.05. Differentially expressed genes were assigned to different Gene Ontology categories using the DAVID software (http://david.abcc.ncifcrf.gov). The microarray data from this study have been submitted to the NCBI Gene Expression Omnibus (http://www.ncbi.nlm.nih.gov/geo) under accession number GSE43035.

### ChIP

It was performed as previously described (17) with minor modifications. Briefly, 10^8^ H295R/TR SF-1 cells (pretreated or not with doxycycline for 3 days) were fixed with 1% formaldehyde added to the culture medium for 10 min at room temperature. After quenching with 0.25 M glycine, cells were washed with phosphate buffered saline and sequentially with Lysis Buffer I [50 mM HEPES–KOH (pH 7.5), 140 mM NaCl, 1 mM EDTA, 10% glycerol, 0.5% NP-40, 0.25% Triton X-100, containing protease inhibitors], II [10 mM Tris–HCl (pH 8.0), 200 mM NaCl, 1 mM EDTA, 0.5 mM EGTA, containing protease inhibitors] and III [10 mM Tris–HCl (pH 8.0), 100 mM NaCl, 1 mM EDTA, 0.5 mM EGTA, 0.1% Na-deoxycholate, 0.5% N-lauroylsarcosine, supplemented with protease inhibitors] and sonicated for 20 cycles at 20% amplitude with a Branson sonifier to shear chromatin to a final average size of ∼200 bp. After centrifugation to pellet debris, the supernatant was incubated overnight at 4°C on a rotating wheel with anti SF-1 (#07-618 from Millipore or PP-N1665-00 from R&D Systems) or anti NRSF/REST (#07-579 from Millipore) antibodies adsorbed to immunomagnetic protein A/protein G beads (Dynabeads, Invitrogen). Beads carrying the immunoprecipitated chromatin were washed five times with RIPA buffer [50 mM HEPES–KOH (pH 7.5), 500 mM LiCl, 1 mM EDTA, 1% NP-40, 0.7% Na-deoxycholate] and once with TE containing 50 mM NaCl. DNA was eluted by incubation with elution buffer [50 mM Tris–HCl (pH 8.0), 10 mM EDTA, 1% SDS] at 65°C for 15 min, and cross-linking reversal was performed by incubation at 65°C overnight. We systematically checked for successful immunoprecipitation of SF-1 in each sample (Supplementary Figure S1A). DNA was then purified by RNAse A treatment and proteinase K digestion, phenol-chloroform extracted, precipitated, resuspended in 10 mM Tris–HCl (pH 8.0) and quantified on a Qubit fluorometer (Invitrogen).

### High-throughput sequencing and data analysis

Immunoprecipitated DNA were sequenced on an Illumina platform, and reads passing quality control criteria were mapped to the human genome (version hg19) using ELAND v2 software. SF-1-binding sites were identified using the CisGenome v2 SeqPeak algorithm (18) from triplicate samples for each SF-1 dosage (basal or increased) condition and input DNA as control, using the following parameters: read extension length = 150, local rate window = 10 kb, local rate cutoff = 1e-005. The Sole-Search web tool (19) was used to annotate the position of SF-1-binding peaks in relationship to gene localization and to import their sequence for further motif searching using the MEME suite (20). The within-gene mode in the Sole-Search Location Analysis Tool was used to assign binding sites to genes. Overlaps between gene groups were identified using the GeneVenn software (http://genevenn.sourceforge.net). To calculate the significance of overlap between gene sets, the chi-square test with Yates’ correction was used, considering that 21 679 genes were present in total on the HG-U133 Plus 2.0 arrays and 19 889 on the Human Gene 1.0 ST arrays. Formaldehyde-Assisted Isolation of Regulatory Elements (FAIRE) sequencing was performed as described (21). High-throughput sequencing data are deposited in Gene Expression Omnibus under accession number GSE44224.

### Electrophoretic mobility shift assay

It was performed as previously described (12). The sequence of probes used for electrophoretic mobility shift assay (EMSA) was as follows: SF-1-binding site (mouse Müllerian inhibiting substance promoter), CGTCCCTCAAGGTCACCTTC; NRSF/REST binding site, CTTCAGCACCACGGACAGCC; CRE (cAMP-response element from rat somatostatin promoter), CTCCTTGGCTGACGTCAGAGAGAGAG. Supershift was performed using rabbit anti SF-1 polyclonal antibody (#07-618, Millipore; 1 µg) or non-immune rabbit immunoglobulin Gs (Sigma-Aldrich) at the same concentration as a control.

### Immunoprecipitation

The 3 × 10^7^ H295R/TR SF-1 cells were resuspended in 800 μl of buffer A [10 mM HEPES–KOH (pH 7.5), 1.5 mM MgCl_2_, 10 mM KCl, 0.1 mM EDTA, 0.2 mM EGTA, 0.2 mM PMSF plus protease inhibitors] and allowed to swell on ice for 15 min. Then 25 μl of 20%NP-40 were added, and cells were vortexed. After centrifugation, the pellet was resuspended in 300 μl of buffer C [20 mM HEPES–KOH (pH 7.5), 20% glycerol, 420 mM NaCl, 1.5 mM MgCl_2_, 0.2 mM EDTA, 0.2 mM PMSF plus protease inhibitors] and incubated for 30 min on ice. After centrifugation, the supernatant fraction was used for coimmunoprecipitation after adjusting the salt concentration to 150 mM. The supernatant was incubated with 3 μg of preimmune rabbit immunoglobulin G (Abcam) or antibodies directed against SF-1 (#07-618 from Millipore) or NRSF/REST (#07-579 from Millipore) overnight at 4°C on a rotating wheel. Twenty-five microliters of Protein A magnetic beads (New England Biolabs) were then added and incubated for 1 h at 4°C. The antibody-antigen-beads complex were washed four times with a buffer containing 20 mM HEPES–KOH (pH 7.5), 150 mM NaCl, 0.5 mM EDTA, 0.5% NP-40 and eluted in Laemmli loading buffer at 70°C. After denaturation at 100°C for 5 min, immunoblot analysis was performed using the same antibodies used for immunoprecipitation.

### Transient transfection

HeLa cells were transfected in 12-well plates using JetPEI (Polyplus Transfection), according to the manufacturer’s protocol. The NRSF/REST luc reporter harbors one NRSF/REST binding site cloned in the *KpnI-XhoI* sites of the pGL2 promoter vector. cDNAs encoding wild-type, L451A/L452A (AF-2 mut) and G35E/R92Q (DBD mut) SF1 were cloned in the *HindIII-BamHI* sites of the pcDNA4/TO vector. Wild-type and mutant SF-1 proteins were all expressed at similar levels in transfected HeLa cells (Supplementary Figure S1B). A vector encoding *Renilla* luciferase was cotransfected for normalization of transfection efficiency. Fourty-eight hours after transfection, cells were lysed, and firefly/*Renilla* luciferase activities were measured using the Dual Luciferase assay (Promega).

## RESULTS

### SF-1 dosage-dependent regulation of distinct categories of genes in adrenocortical cancer cells

To identify the genes regulated by SF-1 in human adrenocortical cancer cells, we performed knockdown and overexpression experiments in the H295R/TR SF-1 cells, a subclone of the H295R cell line where SF-1 overexpression can be induced in a doxycycline-dependent manner (12). Efficient SF-1 knockdown was obtained by synthetic siRNA electroporation (Supplementary Figure S1C). Eighty-three genes were upregulated and 125 genes were downregulated by SF-1 siRNA compared with cells transfected with control siRNA (Supplementary Table S1). On the other hand, SF-1 overexpression (Supplementary Figure S1C) produced upregulation of 184 genes and downregulation of 91 genes in H295R cells (Supplementary Table S1). Remarkably, the categories of genes whose expression was affected by SF-1 knockdown and genes regulated by SF-1 overexpression only marginally overlap. Genes negatively regulated by SF-1 knockdown (activated by SF-1) are enriched in genes involved in lipid and steroid metabolism, Ras guanyl-nucleotide exchange factor activity and cytoskeleton function, whereas genes positively regulated by SF-1 knockdown (repressed by SF-1) are enriched in genes involved in angiogenesis, cell proliferation and apoptosis, interaction with the extracellular matrix, Notch and TGFβ signaling (Table 1; full lists of enriched Gene Ontology categories for genes regulated by SF-1 knockdown are shown in Supplementary Table S2). Conversely, genes repressed by SF-1 overexpression are enriched in genes involved in cell adhesion, angiogenesis, ion transport and apoptosis, whereas genes positively regulated by SF-1 overexpression are enriched in genes involved in angiogenesis, lipid and steroid metabolism, ion transport (Table 1; full lists of enriched Gene Ontology categories for genes regulated by SF-1 overexpression are shown in Supplementary Table S2).

**Table 1.** Gene categories modulated by SF-1 knockdown and overexpression in H295R cells

Downregulated by SF-1 knockdown	Upregulated by SF-1 knockdown	Downregulated by SF-1 overexpression	Upregulated by SF-1 overexpression
Lipid and steroid metabolism	Angiogenesis	Angiogenesis	Angiogenesis
Ras guanyl-nucleotide exchange factor activity	Cell prolifetration	Apoptosis	Lipid and steroid metabolism
Cytoskeleton function	Apoptosis	Cell adhesion	Ion transport
	Interaction with the extracellular matrix	Ion transport	
	Notch signaling		
	TGFβ signaling		

**Figure 1.**
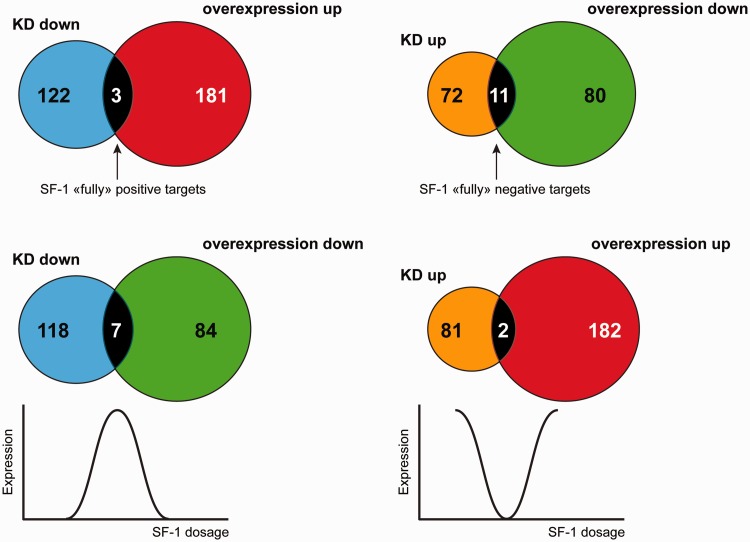
Comparison of genes regulated positively and negatively by SF-1 knockdown and overexpression in H295R cells. Blue circles denote genes downregulated by SF-1 knockdown. Red circles denote genes upregulated by SF-1 overexpression. Orange circles denote genes upregulated by SF-1 knockdown. Green circles denote genes downregulated by SF-1 overexpression. The number of common genes is indicated in the intersection between circles. SF-1 ‘fully’ positive and negative target genes are downregulated by SF-1 knockdown/upregulated by its overexpression and upregulated by SF-1 knockdown/downregulated by its overexpression, respectively. The expression of some SF-1 target genes follows a bell-shaped pattern according to dosage of the factor (curves at the bottom of the figure).

Comparison of genes regulated (both up- and down-) by SF-1 knockdown and overexpression revealed that only a small subset was common among the different experimental conditions (Figure 1). Only three genes can be considered SF-1 ‘fully’ positive targets, as they are downregulated by SF-1 knockdown and upregulated by SF-1 overexpression. Conversely, 11 other genes are SF-1 ‘fully’ negative targets, as their expression is upregulated by SF-1 knockdown and downregulated by SF-1 overexpression. It is interesting to notice that the expression of some SF-1 target genes follows a coordinately regulated bell-shaped pattern according to factor dosage, with one group being downregulated and the other being upregulated both by SF-1 knockdown and overexpression (Supplementary Table S3). Altogether, these findings show that distinct categories of genes are regulated according to SF-1 dosage in H295R cells.

### The localization of SF-1 genomic-binding sites differs in conditions of basal and increased dosage and is correlated with changes in gene expression

**Figure 2.**
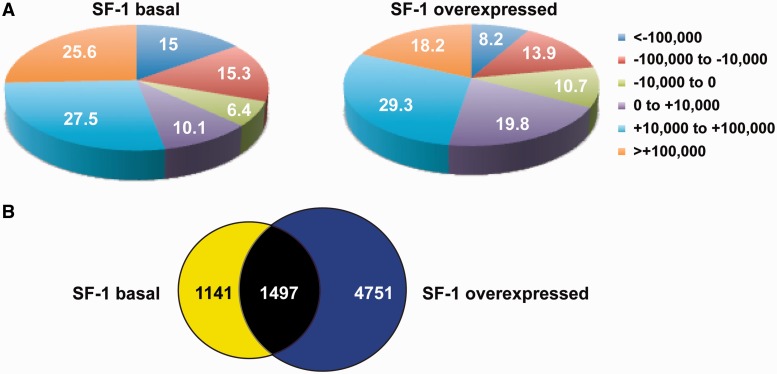
Relationship of SF-1-binding sites to the localization of genes in H295R cells in basal and overexpression conditions. (**A**) Pie charts showing the distribution of SF-1-binding sites in relationship to transcription start sites in basal (left) and increased (right) factor expression conditions. Legend indicates distance from transcription start sites in base pairs. The percentage of SF-1-binding sites in each distance group is indicated. (**B**) Overlap between genes in whose proximity (≤100 kb) SF-1-binding sites were detected. Yellow circle denotes basal SF-1 dosage. Blue circle denotes increased SF-1 dosage.

**Figure 3.**
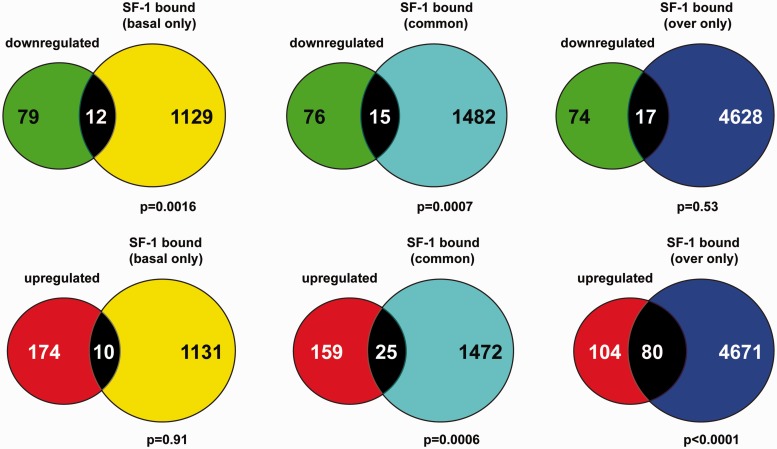
Correlation between SF-1 binding and gene regulation. Green circles denote genes downregulated by SF-1 overexpression. Red circles denote genes upregulated by SF-1 overexpression. Yellow circles denote genes bound by SF-1 only in basal expression conditions. Blue circles denote genes bound by SF-1 only when overexpressed. Pale blue circles denote genes bound by SF-1 both in basal and overexpression conditions. The number of common genes is indicated in the intersection between circles. The significance value for each association is indicated (chi-square with Yates’ correction test).

To understand the bases for differential gene regulation by varying SF-1 dosage, we identified genome-wide binding sites of SF-1 to chromatin of H295R cells in conditions of basal expression and overexpression (Figure 2). In triplicate experiments, SF-1 bound to 4393 sites in basal expression conditions, whereas it bound to 11 910 sites when overexpressed. The majority (83.5%) of SF-1-binding sites in basal conditions was located distal (>10 kb) to gene boundaries, whereas when SF-1 was overexpressed, its association to gene-proximal sites (<10 kb) increased (30.5%) (Figure 2A and Supplementary Figure S2A and B). In particular, the percentage of SF-1-binding sites in close proximity of transcription start sites (−1000 to + 1000 bp) was increased more than 3-fold when SF-1 was overexpressed. In basal expression conditions, the percentage of SF-1-binding sites lying in gene deserts (distant >100 kb from gene boundaries) totaled 40.6%, whereas it was reduced to 26.4% when SF-1 was overexpressed (Supplementary Figure S2C). Remarkably, only 545 SF-1-binding sites were common to the basal and overexpression conditions (Supplementary Table S4). However, the overlap between genes in whose proximity SF-1 bound in both conditions was more important: when considering SF-1 binding within <100 kb of transcription start sites, it bound in the proximity of 2638 unique genes in basal expression conditions, whereas this number increased to 6248 when SF-1 was overexpressed. In all, 1497 genes were common between these two data sets (Figure 2B and Supplementary Table S4). We next examined the relationship between genes in whose proximity SF-1 bound in basal and overexpression conditions and genes that are differentially regulated by SF-1 dosage (Figure 3). No significant overlap existed between genes in whose proximity SF-1 bound only in conditions of basal expression and genes that were upregulated following SF-1 overexpression in H295R cells (*P=*0.91). Conversely, a significant association existed (*P* = 0.0006) between genes in whose proximity SF-1 bound both in basal and overexpression conditions and genes upregulated following SF-1 overexpression. The highest significant association (*P* < 0.0001) was between genes in whose proximity SF-1 bound only when overexpressed and genes upregulated following its overexpression. When considering genes downregulated following SF-1 overexpression, a significant association existed with genes in whose proximity SF-1 bound only in basal expression conditions (*P=*0.0016), and with genes commonly bound both by basal and overexpressed SF-1 (*P=*0.0007), whereas no significant association existed with genes in whose proximity SF-1 bound only in overexpression conditions (*P=*0.53). Altogether, these data show that differential SF-1 binding at basal and increased expression levels has a significant impact on gene expression in H295R cells.

### A subset of SF-1-binding sites colocalizes with newly accessible sites in H295R cells chromatin

To investigate the relationship of SF-1-binding sites with accessible sites in chromatin, we used FAIRE-seq. Using this technique, nucleosome-depleted DNA sequences are preferentially identified that overlap with regulatory sites in the genome (22). In H295R cells expressing basal levels of SF-1, 34 421 FAIRE sites were identified, while 31 217 were present in cells overexpressing SF-1, with 18 703 FAIRE sites being common between both conditions. These data show that FAIRE sites undergo substantial rearrangements after SF-1 overexpression in H295R cells. FAIRE sites were preferentially localized inside or in the proximity of genes, both in SF-1 basal expression and overexpression conditions (Supplementary Table S4). Remarkably, 1008 SF-1-binding sites identified in SF-1 overexpression conditions overlapped with FAIRE sites present only when SF-1 was overexpressed. This indicates that SF-1 is able to associate with newly accessible sites in H295R cells chromatin on its overexpression and may have a role in their establishment.

### Enrichment of specific DNA sequences in SF-1-binding sites in H295R cells

**Figure 4.**
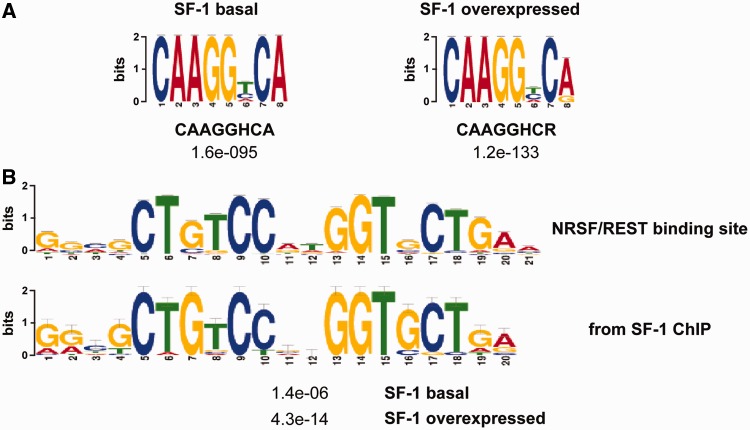
Enrichment of SF-1 and NRSF/REST binding sequences in SF-1-binding sites in H295R cells. (**A**), A motif closely matching the sequence to which SF-1 binds *in vitro* is significantly enriched in SF-1-binding sites, both at basal (left) and increased (right) SF-1 dosage. (**B**), A motif (top) closely matching the NRSF/REST binding sequence is significantly enriched in SF-1-binding sites.

Sequence enrichment analysis of SF-1-binding sites using several algorithms (20) revealed that the consensus SF-1-binding sequence identified *in vitro* (23) or close variants of it are significantly enriched in SF-1-binding sites in the chromatin of H295R cells both when the factor is expressed at basal levels and in overexpression conditions (Figure 4A). Unexpectedly, a sequence matching the consensus binding sequence for transcription factor NRSF/REST (24) was also significantly enriched in SF-1-binding sites in both basal and high SF-1 dosage conditions (Figure 4B). Importantly, the presence of SF-1 and NRSF/REST consensus-binding sites in sequences bound by SF-1 nearly always appears mutually exclusive, as only minimal overlap existed between SF-1-binding sites containing the SF-1 consensus sequence and those containing the NRSF/REST consensus sequence (1.2% for basal SF-1 dosage and 1% for increased SF-1 dosage).

FAIRE sites in H295R cells were significantly enriched in sequences matching a variety of transcription factors binding sites, including the SF-1 sequence at a high significance level (*P=*7.3e-300 in basal SF-1 expression conditions and *P=*1.7e-433 when SF-1 was overexpressed) (Supplementary Figure S3). CTCF sites were also frequently represented in FAIRE sites (*P=*4.8e-261 in basal SF-1 expression conditions and *P=*1.8e-60 when SF-1 was overexpressed) consistently with previous reports in other cell lines (22). Remarkably, sequences in SF-1-binding sites in overexpression conditions overlapping with FAIRE sites present only when SF-1 was overexpressed were significantly enriched in SF-1 (*P=*1.7e-07) and NRSF/REST (*P=*2.9e-09) but not CTCF (*P=*ns) sites.

### A dynamic interplay between SF-1 and NRSF/REST in the regulation of gene expression

**Figure 5.**
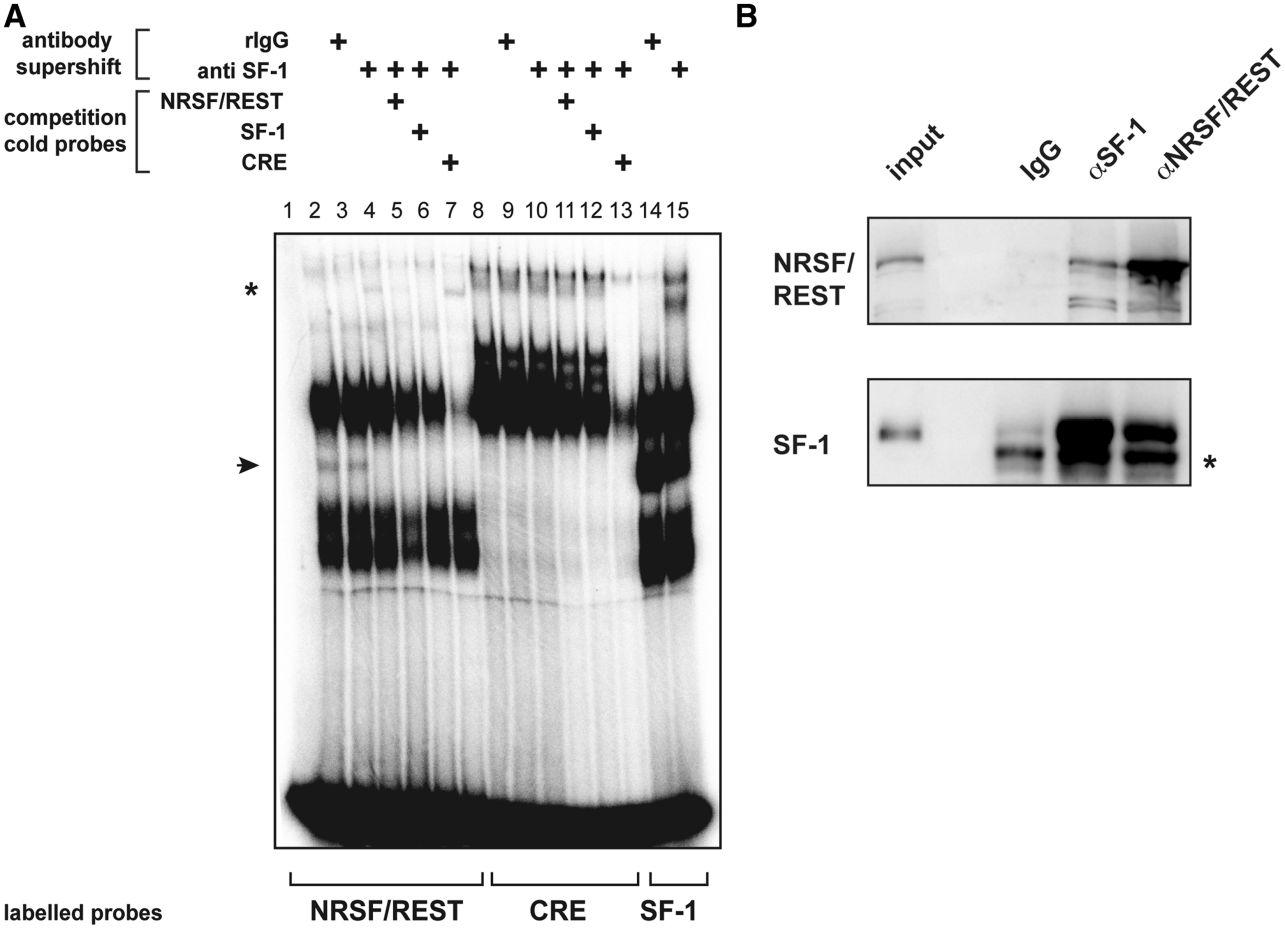
Interaction of SF-1 with NRSF/REST. (**A**) EMSA showing interaction of SF-1 with the NRSF/REST sequence. Lane 1, free NRSF/REST labeled probe; lanes 2–7, binding of H295R nuclear extracts to the NRSF/REST labeled probe; lanes 8–13, binding of H295R nuclear extracts to the CRE labeled probe; lanes 14–15, binding of H295R nuclear extracts to the labeled SF-1 probe. In lanes 3, 9 and 14, rabbit immunoglobulin G was added, and in lanes 4–7, 10–13 and 15, the anti SF-1 antibody was added to the binding reaction, respectively. Cold NRSF/REST, SF-1 and CRE probes were also added to binding reactions in lanes 5 and 11, 6 and 12, 7 and 13, respectively. An intermediate mobility complex (arrowhead) was formed, which comigrates with the complex formed by the same extracts on the SF-1 consensus binding sequence (lane 14). This complex was supershifted (asterisk) by an anti SF-1 antibody (lane 4), but not by non-immune rabbit immunoglobulin Gs (lane 3). The complex did not form on an unrelated DNA sequence (CRE, lanes 8–12). An overexposed image of the same EMSA experiment is shown in Supplementary Figure S5. (**B**) SF-1 can be co-immunoprecipitated with NRSF/REST from H295R nuclear extracts. Immunoprecipitates were analyzed by immunoblotting for the presence of SF-1 and NRSF/REST. Input, 1:10 input extract; IgG, rabbit immunoglobulin G; αSF-1, anti SF-1 antibody; αNRSF/REST, anti NRSF/REST antibody. The asterisk indicates a non-specific band present in the SF-1 immunoblotting.

The unexpected enrichment of NRSF/REST binding sequences in SF-1-binding sites prompted us to analyze the functional interactions between those factors in the regulation of gene expression in H295R cells. Importantly, NRSF/REST sites were also significantly enriched in SF-1-binding sites identified using a different anti SF-1 antibody for ChIP (*P=*8.4e-08). We mapped the distribution of NRSF/REST binding sites in H295R cells by ChIP-seq. NRSF/REST bound to a similar number of sites in conditions of basal (5322 sites) and increased (5058 sites) SF-1 expression, respectively. Although substantial redistribution of NRSF/REST binding sites occurred on increase of SF-1 dosage, with 3471 binding sites overlapping in conditions of basal and increased SF-1 dosage, their localization in relationship to gene positions only underwent minimal changes, with the majority of sites being intragenic (39.4% both under basal and increased SF-1 dosage; Supplementary Figure S4). Sequence enrichment analysis found the consensus NRSF/REST binding sequence as highly enriched both in conditions of basal (*P=*7.5e-306) and increased (*P=*1.2e-300) SF-1 expression, while no sequence related to the SF-1 consensus-binding site was detected as significantly enriched. Remarkably, 109 (2% of total NRSF/REST binding sites; 2.5% of total SF-1-binding sites) and 196 (3.8% of total NRSF/REST binding sites; 1.6% of total SF-1-binding sites) NRSF/REST and SF-1-binding sites overlapped in conditions of basal and increased SF-1 expression, respectively. Motif searching in those binding sites identified the NRSF/REST consensus sequence in 105 of 109 cases (96.3%; basal SF-1 dosage) and 150 of 196 cases (76.5%; increased SF-1 dosage), whereas the SF-1 consensus sequence was present in only 19 of 109 cases (17.4%; basal SF-1 dosage) and 30 of 196 cases (15.3%; increased SF-1 dosage), respectively. These differences are extremely significant (*P* < 0.0001 in both cases, Fisher’s exact test) and strongly suggest that the presence of NRSF/REST, but not SF-1, consensus sequences is preferentially required for interaction of SF-1 with the genomic-binding sites that it shares with NRSF/REST in H295R cells. Consistent with this hypothesis, when binding of H295R nuclear extracts to a NRSF/REST sequence was assayed in an EMSA, an intermediate mobility complex formed, which co-migrated with the complex formed on the SF-1 consensus-binding sequence (Figure 5A). This complex was supershifted by an antibody specifically recognizing SF-1, whereas non-immune rabbit immunoglobulin Gs had no effect on the mobility of the complex. Moreover, the anti–SF-1 supershifted complex was competed both by cold NRSF/REST and SF-1 sequences but not by an unrelated (cAMP-response element, CRE) cold sequence. Conversely, the complex did not form on the CRE sequence. Those SF-1-containing complexes are more evident in an overexposed image of the same EMSA experiment shown in Supplementary Figure S5. Furthermore, SF-1 and NRSF/REST could be co-immunoprecipitated from H295R nuclear extracts (Figure 5B).

**Figure 6.**
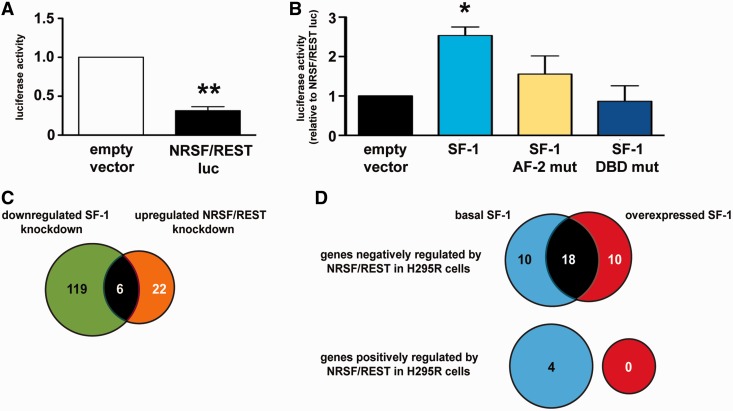
Modulation of NRSF/REST-dependent gene expression by SF-1 dosage. (**A**) The NRSF/REST binding sequence cloned upstream of a basal promoter represses its expression in HeLa cells. **P = 0.0025, Wilcoxon signed rank test. (**B**) Co-transfection of wild-type, but not AF-2 (L451A/L452A; AF-2 mut) and DNA-binding domain (G35E/R92Q; DBD mut) SF-1 mutants relieved transcriptional repression imparted by the NRSF/REST sequence. *P < 0.05, ANOVA with Dunnett’s multiple comparison test. (**C**) Comparison of the sets of genes downregulated by SF-1 knockdown (positively regulated by SF-1) and upregulated by NRSF/REST knockdown (negatively regulated by NRSF/REST). (**D**) Comparison of NRSF/REST-regulated genes in H295R cells in conditions of basal and increased SF-1 dosage. Blue circles denote genes regulated by NRSF/REST in the presence of basal SF-1 dosage; red circles denote genes regulated by NRSF/REST in the presence of increased SF-1 dosage. The number of common genes is indicated in the intersection between circles.

Based on these results, we examined whether a functional interaction exists between SF-1 and NRSF/REST in the regulation of gene expression. As shown before (25), in non-neuronal cells (HeLa), the NRSF/REST binding sequence confers significant transcriptional repression activity when cloned upstream a basal promoter (NRSF/REST luc; Figure 6A). Wild-type SF-1 could significantly relieve transcriptional repression of NRSF/REST luc, whereas both an AF-2 (L451A/L452A) (12) and a DNA-binding domain (G35E/R92Q) (26) SF-1 mutants were inactive (Figure 6B). These data show that SF-1 transcriptional activity, either directly or interfering with repression by NRSF/REST, and DNA-binding properties are both required to relieve transcriptional repression imparted by NRSF/REST. We then looked at the impact of varying SF-1 dosage on NRSF/REST-regulated gene expression in H295R cells. In this cell line, as assessed by knockdown experiments (Supplementary Figure S1D), NRSF/REST regulated negatively the expression of 28 genes and positively the expression of 4 genes in basal SF-1 expression conditions (Supplementary Table S5). NRSF/REST target genes were enriched in genes involved in ion trafficking, membrane function and, surprisingly, also in steroidogenesis (Supplementary Table S5). It is important to notice that SF-1 and NRSF/REST share a highly significantly overlapping (*P* < 0.0001, Fisher’s exact test) subset of target genes, including the steroidogenic genes *CYP21A2* and *CYP19A1* (Figure 6C and Supplementary Table S5). Strikingly, when SF-1 was overexpressed, an overlap of 18 genes negatively regulated by NRSF/REST existed with genes negatively regulated by this factor in the presence of a basal SF-1 dosage, while 10 were specific only to the basal SF-1 dosage and 10 to the overexpressed SF-1 condition (Figure 6D and Supplementary Table S5). In particular, NRSF/REST repression of *CYP21A2* and *CYP19A1* was abrogated by an increased SF-1 dosage. Moreover, the NRSF/REST positive regulatory action was completely impaired by overexpressed SF-1 (Figure 6D).

## DISCUSSION

For several transcription factors, gene dosage is a critical determinant of their biological function, a paradigm case being represented by Oct-3/4 dosage in mouse embryonic stem cells (27). Among nuclear receptors, the role of androgen receptor and estrogen receptor-α amplification and overexpression in prostate and breast cancer progression, respectively, is well known (28,29). The first indications about the importance of SF-1 dosage for its biological function came from genetic inactivation experiments in mice. Although *Nr5a1* homozygote inactivation causes complete absence of adrenal glands and gonads and spleen defects (4,5,30), *Nr5a1* heterozygote mice have hypoplastic adrenals and gonads, in the presence of decreased corticosterone and increased (especially after stress) ACTH plasma levels (8), also displaying a deficient compensatory adrenal growth following unilateral adrenalectomy (9). It is particularly remarkable that different tissues appear to possess different thresholds of sensitivity to *Nr5a1* gene dosage, as shown by the finding that SF-1 re-expression in the *Nr5a1* null background rescued gonadal and spleen defects, but failed to rescue adrenal development (31). This different sensitivity to *NR5A1* dosage also exists in humans, where heterozygote *NR5A1* mutations are most commonly associated with gonadal, but not adrenal, defects (10,11). Conversely, SF-1 overexpression is associated with increased adrenocortical tumor cell proliferation, development of adrenocortical tumors in mice and is a widespread finding in pediatric adrenocortical tumors, whereas it confers a more severe prognosis to adult adrenocortical cancers (12–14). Consistent with the relevant phenotypic effects of different SF-1 dosages, our work shows that different SF-1 levels in the H295R adrenocortical tumor cell line differentially regulate the expression of numerous genes, which are involved in a variety of biological processes [reviewed in (32)]. Remarkably, the functions of genes regulated by different SF-1 dosages only partially overlap, with mainly distinct gene categories being regulated by basal and overexpressed SF-1 levels (Table 1). SF-1 target genes are implicated in functions as diverse as ion transport, lipid metabolism, angiogenesis, cell survival and control pivotal pathways such as TGFβ and Notch signaling. As a consequence, only a limited set of genes is commonly regulated by SF-1 both in basal and overexpression conditions. These data significantly extend the list of SF-1 targets identified by previous studies in H295R cells (33–35) and suggest that fine regulation of SF-1 dosage is a critical determinant of its action during adrenal development, function and tumorigenesis. It is also interesting to notice that the expression of some SF-1 target genes follows a bell-shaped pattern according to factor dosage, with one group being downregulated and the other being upregulated both by SF-1 knockdown and overexpression (Supplementary Table S3). *NOV,* encoding a secreted pro-apoptotic factor for adrenocortical cancer cells (36), is an example of those genes that are tightly regulated according to SF-1 dosage.

To better understand the bases of the peculiar mode of gene expression regulation by SF-1, we identified its chromatin-binding sites on a genome-wide scale using ChIP-seq both in basal and in overexpression conditions. Under basal expression conditions, SF-1 bound to >4000 sites in H295R cells, which nearly tripled when SF-1 was overexpressed. Similarly to many other nuclear receptors (37), the majority of SF-1 binding sites was in both cases localized distal to genes. However, when SF-1 was overexpressed, its association to gene-proximal sites markedly increased (30.5 versus 16.6% in basal expression conditions). It is interesting to notice that the percentage of genes commonly bound by SF-1 in basal and overexpressed conditions exceeded the number of common binding sites, suggesting that SF-1-regulated genes have alternative SF-1-binding sites. Our data show that at least part of the regulation operated by SF-1 on its target genes can be explained by differential SF-1 binding at basal or increased expression levels. Particularly, a high correlation existed between the localization of SF-1-binding sites present only when the factor was overexpressed and genes upregulated selectively in that condition, consistently with the classical SF-1 function as a transcriptional activator. Similarly, the correlation between downregulation of certain genes when SF-1 was overexpressed in the absence of SF-1 binding, which was present only in conditions of basal factor dosage, can be interpreted as a withdrawal of transcriptional activation. On the other hand, it is interesting to notice that a correlation also existed between downregulation of a subset of genes and SF-1 binding in their proximity in both basal and overexpression conditions. This suggests that in certain cases, SF-1 may directly repress gene expression, consistently with previous reports (38,39) and findings for other nuclear receptors classically considered as pure transcriptional activators (40). Finally, indirect mechanisms (e.g. through regulation of the expression of other transcription factors) or long-range effects are likely to account for the regulatory function of different SF-1 dosages on genes in whose proximity the factor does not directly bind.

We also studied the relationship of SF-1-binding sites with accessible sites in chromatin, identified by FAIRE-seq (22), which are the hallmark of regulatory regions in the genome. In H295R cells, FAIRE sites are remarkably highly enriched for a sequence highly similar to the sequence bound by SF-1 *in vitro* (23), together with other transcription factor (including CTCF, known to bind to insulator regions) binding sites. This is consistent with results from the ENCODE consortium showing that cell-type selective open chromatin regions harbor DNA sequence motifs bound by master regulators of cellular identity (22). The sequence bound by SF-1 in living cells matches almost completely the consensus sequence defined *in vitro*. However, no enrichment is present for a pyrimidine lying just 5′ to the cytosine upstream the nuclear receptor half-site, which makes crucial contacts with the C-terminal extension A box in the structures of SF-1 and its close homologue LRH-1 DBDs complexed with their bound sequences (41,42). These data suggest that SF-1-cognate DNA interaction is probably stabilized *in vivo* by interaction with the chromatin context or with other transcriptional complexes. It is also remarkable that SF-1 overexpression in H295R cells induced substantial modifications in the localization of their FAIRE sites. In particular, a subset of SF-1 binding sites was co-localized with FAIRE sites only present when SF-1 was overexpressed. This suggests that SF-1 may be associated with chromatin-remodeling complexes and actively participate in formation of accessible sites in chromatin, similarly to other nuclear receptors (43).

An unexpected result of our study is represented by the discovery of a functional interaction of SF-1 with the transcription factor NRSF/REST in regulating gene expression in adrenocortical cancer cells. NRSF/REST was identified as a transcriptional repressor that suppresses expression of neural genes in non-neuronal cells (15,16) and is believed to have a fundamental role to restrict neural gene expression to the nervous system. However, recent studies have suggested a broader role for this protein in fine-tuning neural gene expression, rather than as a regulator of neurogenesis or cell fate (44). NRSF/REST represses transcription by binding an extended DNA recognition sequence (24,45) and recruiting a corepressor complex (25). Little is known about the role of NRSF/REST beyond its role in restricting neural identity. Remarkably, a RNAi-based genetic screen identified a role for NRSF/REST as a tumor suppressor (46). In adrenocortical H295R cells, a previous report described a role for NRSF/REST in the regulation of aldosterone and cortisol production by regulating *CYP11B1* and *CYP11B2* expression through repression of the *CACNA1H* gene (47).

Here, we have shown that SF-1 and NRSF/REST functionally interact in regulating gene expression in H295R adrenocortical cancer cells. The NRSF/REST binding sequence was significantly enriched in SF-1-binding sites in H295R cells chromatin, both in basal and increased SF-1 expression conditions. Importantly, enrichment of the NRSF/REST sequence was confirmed in SF-1-binding sites also by ChIP with a different anti-SF-1 antibody. Moreover, that sequence was also enriched in FAIRE sites present only when SF-1 was overexpressed. Mapping of NRSF/REST binding sites in H295R cells by ChIP-seq showed that a small percentage of them overlapped with SF-1-binding sites, both under basal and increased SF-1 expression conditions. SF-1 and NRSF/REST could be co-immunorecipitated from H295R nuclear extracts and among the complexes formed by H295R nuclear extracts with the NRSF/REST sequence in EMSA, an intermediate mobility complex was detectable which contained SF-1, as shown by supershift with an anti-SF-1 antibody, and comigrated with the complex formed by SF-1 on its consensus binding sequence. In the overwhelming majority of cases, SF-1-binding sites mapped by ChIP-seq containing the NRSF/REST sequence did not include a consensus SF-1-binding sequence. Moreover, the NRSF/REST sequence was significantly more enriched than the SF-1 consensus sequence in the binding sites commonly occupied by NRSF/REST and SF-1. Altogether, these findings strongly argue in favor of the possibility for SF-1 to bind to some genomic sites in H295R cells by interacting with the NRSF/REST sequence. This binding is probably stabilized by interaction of SF-1 with the NRSF/REST protein bound to its cognate sequence, as suggested by the fact that the two proteins can be co-immunoprecipitated. Functional experiments are supporting this scenario, as cotransfection of wild-type, but not AF-2 mutant or DBD mutant, SF-1 could relieve repression imparted by the NRSF/REST sequence on a basal promoter.

Knockdown experiments showed that NRSF/REST regulates a small set of genes in H295R cells, both negatively and positively [these latter ones probably through indirect effects; see (45)]. Apart from classical target genes expressed in neuronal cells (ion channels, adhesion molecules) and in H295R cells, NRSF/REST also regulated genes involved in steroidogenesis. SF-1 and NRSF/REST shared a significant proportion of their target genes and SF-1 overexpression reshaped the repertoire of NRSF/REST-regulated genes, which only partially overlapped in conditions of basal and increased SF-1 dosage. SF-1 overexpression completely inhibited the positive effect of NRSF/REST on a subset of genes and relieved repression of *CYP21A2* and *CYP19A1*, two important steroidogenic genes. These data show that NRSF/REST has a novel function in regulating the steroidogenic program in adrenocortical cells and, together with recent reports in other systems (48–50), suggest that it may have a broad role in regulating tissue-specific gene expression programs.

In conclusion, we have shown here that SF-1 regulates distinct categories of genes in adrenocortical cancer cells according to its dosage and that binding of the factor to chromatin sites in conditions of different SF-1 dosage correlates with differential regulation of gene expression. Furthermore, these studies allowed us to discover an unexpected functional interaction of SF-1 with NRSF/REST in regulating steroidogenic gene expression. These data are important to understand the mechanisms of gene expression regulation by altered SF-1 dosages in development and cancer and to develop new therapeutic tools targeting SF-1 and its target genes.

## SUPPLEMENTARY DATA

Supplementary Data are available at NAR Online.

## FUNDING

Institut National du Cancer [2008-045]; Agence Nationale de la Recherche [BSV1 005 01 - BeyondTASKs]; Conseil Général 06; CNRS; Association pour le Recherche sur le Cancer [Projets ARC SFI20111203563]; European Union Seventh Framework Programme [FP7/2007–2013] under grant agreement [n°259735 (ENS@T-CANCER) to E.L.]. Funding for open access charge: Institut National du Cancer.

*Conflict of interest statement*. None declared.

## Supplementary Material

Supplementary Data
