# Supplementary material for: Integrative analysis of SF-1 transcription factor dosage impact on genome-wide binding and gene expression regulation
Source: Nucleic Acids Res. 2013 Aug 1;41(19):8896–907. doi: 10.1093/nar/gkt658 (PMC3799431; doi:10.1093/nar/gkt658)
Supplement: Supplementary Data [file supp_41_19_8896__index.html]

Integrative analysis of SF-1 transcription factor dosage impact on genome-wide binding and gene expression regulation — Integrative analysis of SF-1 transcription factor dosage impact on genome-wide binding and gene expression regulation — Supplementary Data 

# Integrative analysis of SF-1 transcription factor dosage impact on genome-wide binding and gene expression regulation

## 

files

**Files in this Data Supplement:**

- Supplementary Data - pdf file
- Supplementary Data - xls file
- Supplementary Data - xls file
- Supplementary Data - xls file
- Supplementary Data - xls file
- Supplementary Data - xls file
