# Supplementary material for: Integrative analysis of SF-1 transcription factor dosage impact on genome-wide binding and gene expression regulation
Source: Nucleic Acids Res. 2013 Aug 1;41(19):8896–907. doi: 10.1093/nar/gkt658 (PMC3799431; doi:10.1093/nar/gkt658)

Legend to Supplementary Figures

Supplementary Figure 1. SF-1 and NRSF/REST protein levels in H295R/TR SF-1 and HeLa cells. A, SF-1 immunoblot after ChIP in H295R/TR SF-1 cells, both in basal (left) and increased (right) SF-1 expression conditions. Rabbit IgGs (-) were used as negative control for ChIP. Asterisk, IgG heavy chain; arrowhead, SF-1 – specific band. B, Expression of wild-type, AF-2 (L451A/L452A; AF-2 mut) and DNA-binding domain (G35E/R92Q; DBD mut) SF-1 mutants after transfection in HeLa cells. β-tubulin levels are also shown as control. C, SF-1 protein expression after knockdown and overexpression in H295R/TR SF-1 cells. Left, SF-1 expression was measured by immunoblot after nucleofection with control (siC) and SF-1 – specific (siSF-1) siRNAs. β-tubulin levels are also shown as control. Right, SF-1 expression in basal conditions and after doxycycline treatment. β-tubulin levels are also shown as control. D, Knockdown of NRSF/REST protein expression by a specific (siR) siRNA, as compared to a contron (siC) siRNA. β-tubulin levels are also shown as control.

Supplementary Figure 2. Relationship between localization of SF-1 binding sites and gene boundaries. A, Basal SF-1 dosage. B, Increased SF-1 dosage. C, Histogram showing the percentage of SF-1 binding sites in gene deserts (farther than 100 kb from genes) in basal (green) and increased (orange) SF-1 expression conditions.

Supplementary Figure 3. List of the most enriched motifs in H295R FAIRE sites present only in conditions of basal (left) or increased (right) SF-1 dosage.

Supplementary Figure 4. Distribution of NRSF/REST binding sites in H295R cells in conditions of basal and increased SF-1 dosage. 5d, regions between 10 and 100 kb upstream of a refseq gene; 5p2, regions between 2 and 10 kb upstream of a refseq gene; 5p1, regions less than 2 kb upstream of a refseq gene; gene, any exon or intron of a refseq gene; 3p1, regions less than 2 kb downstream of the last exon of a refseq gene; 3p2, regions between 2 and 10 kb downstream of the last exon of a refseq gene; 3d, regions between 10 and 100 kb downstream of the last exon of a refseq gene; gd, gene desert, including regions greater than 100 kb from a refseq gene.

Supplementary Figure 5. Overexposed image of the EMSA experiment shown in Figure 5A.

Supplementary Figure 1

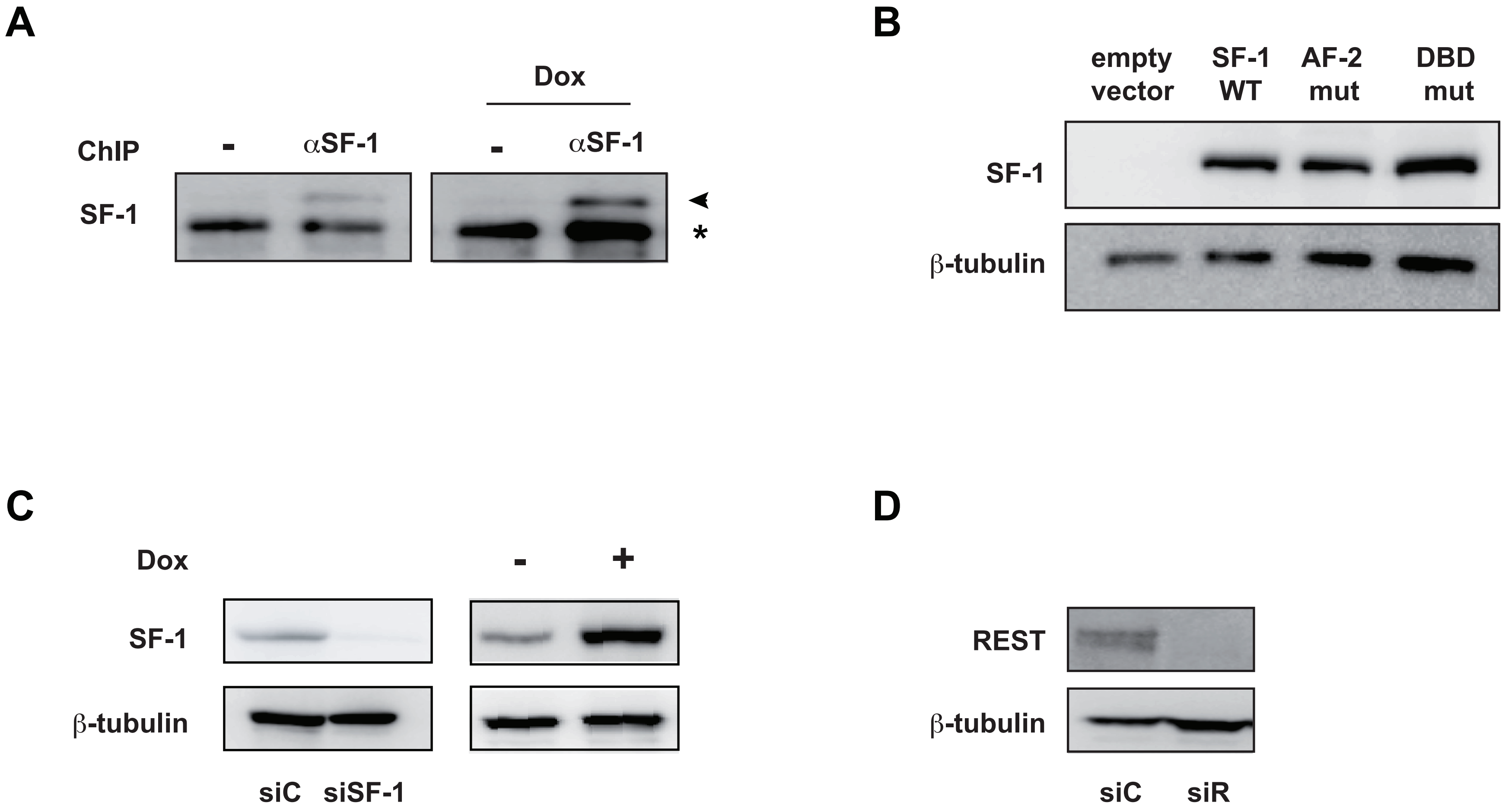

Supplementary Figure 2

A

SF-1 basal

	<100 Kb	-100 to -10 Kb	-10 to -5 Kb	-5 to -1 Kb	-1 Kb to 0	0 to 1 Kb	1 to 5 Kb	5 to 10 Kb	10 to 100 Kb	>100 Kb	Total
binding sites	661	673	93	113	76	112	182	151	1206	1126	**4393**
%	*15*	*15.3*	*2.1*	*2.6*	*1.7*	*2.6*	*4.2*	*3.4*	*27.5*	*25.6*	

16.6 (-10 to -5 Kb through 5 to 10 Kb)

B

SF-1 overexpressed

	<100 Kb	-100 to -10 Kb	-10 to -5 Kb	-5 to -1 Kb	-1 Kb to 0	0 to 1 Kb	1 to 5 Kb	5 to 10 Kb	10 to 100 Kb	>100 Kb	Total
binding sites	977	1650	339	354	580	1027	736	595	3489	2163	**11910**
%	*8.2*	*13.8*	*2.8*	*3*	*4.9*	*8.6*	*6.2*	*5*	*29.3*	*18.2*	

30.5 (-10 to -5 Kb through 5 to 10 Kb)

C

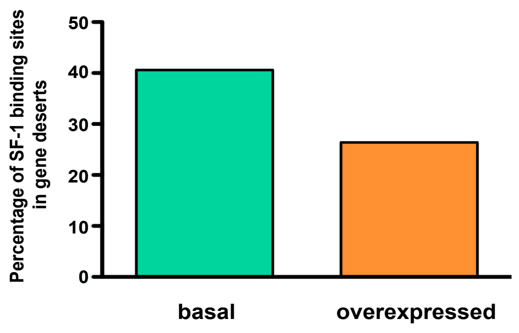

Supplementary Figure 3

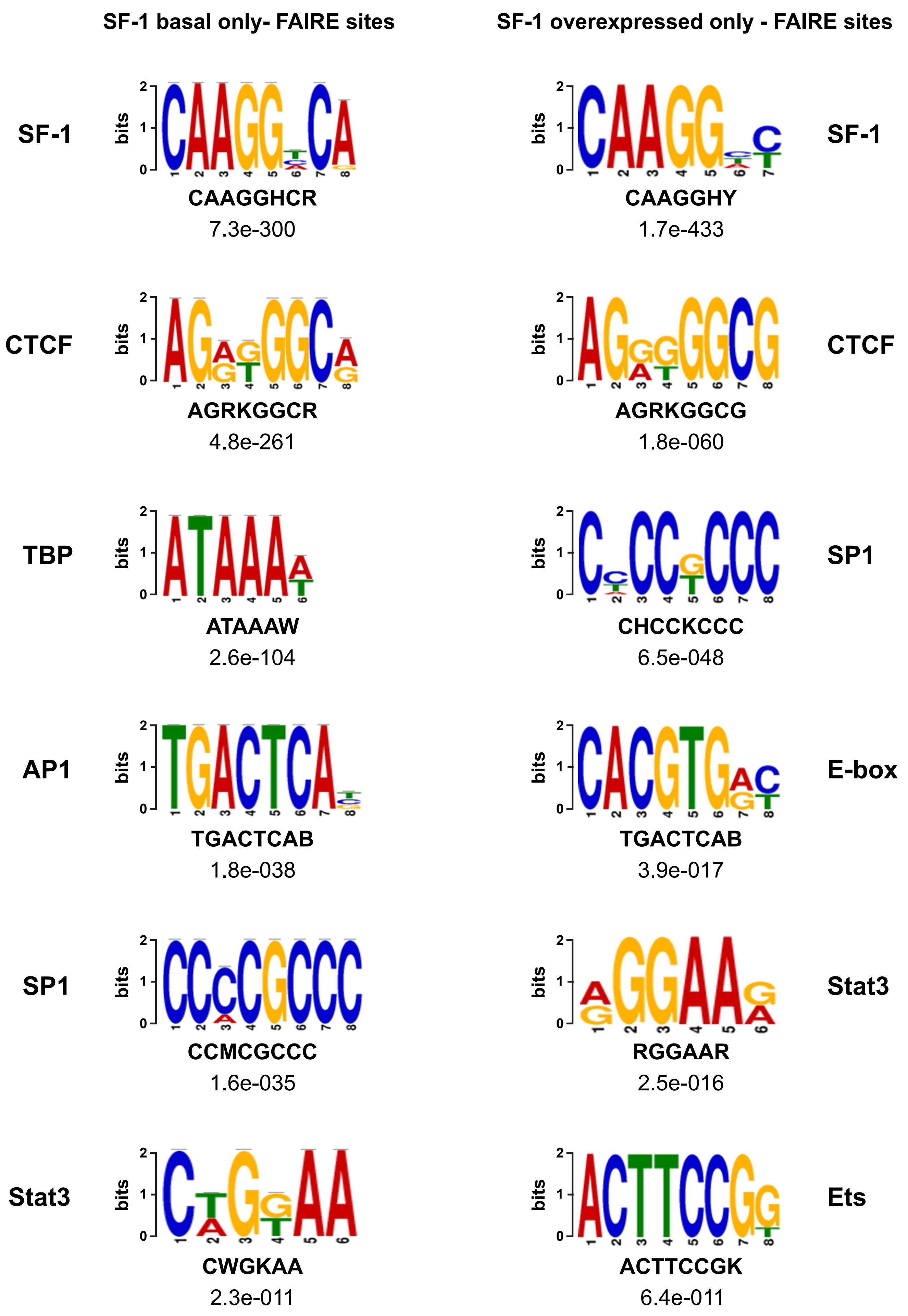

Supplementary Figure 4

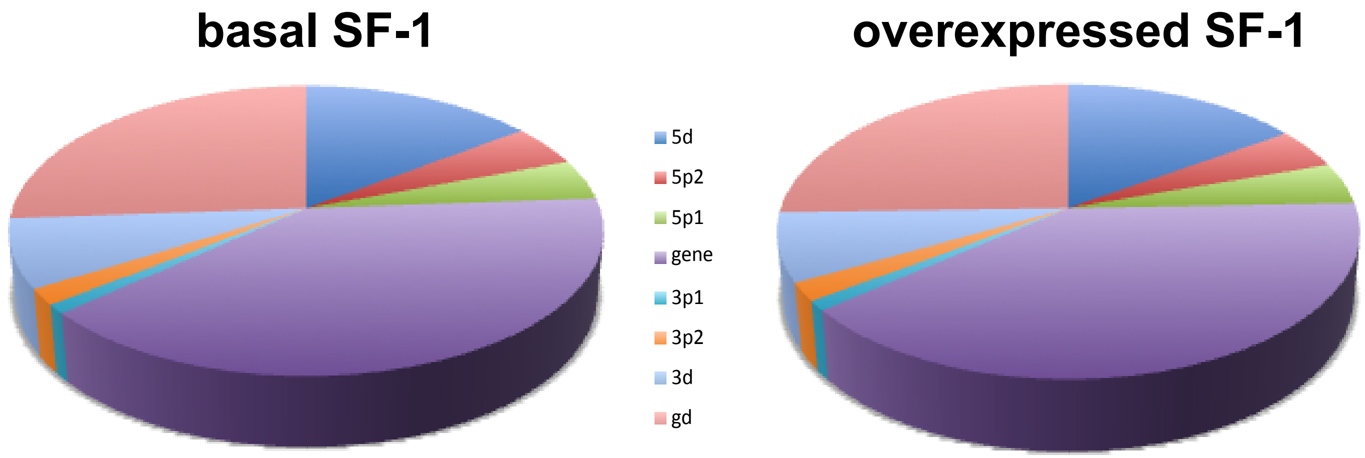

Supplementary Figure 5

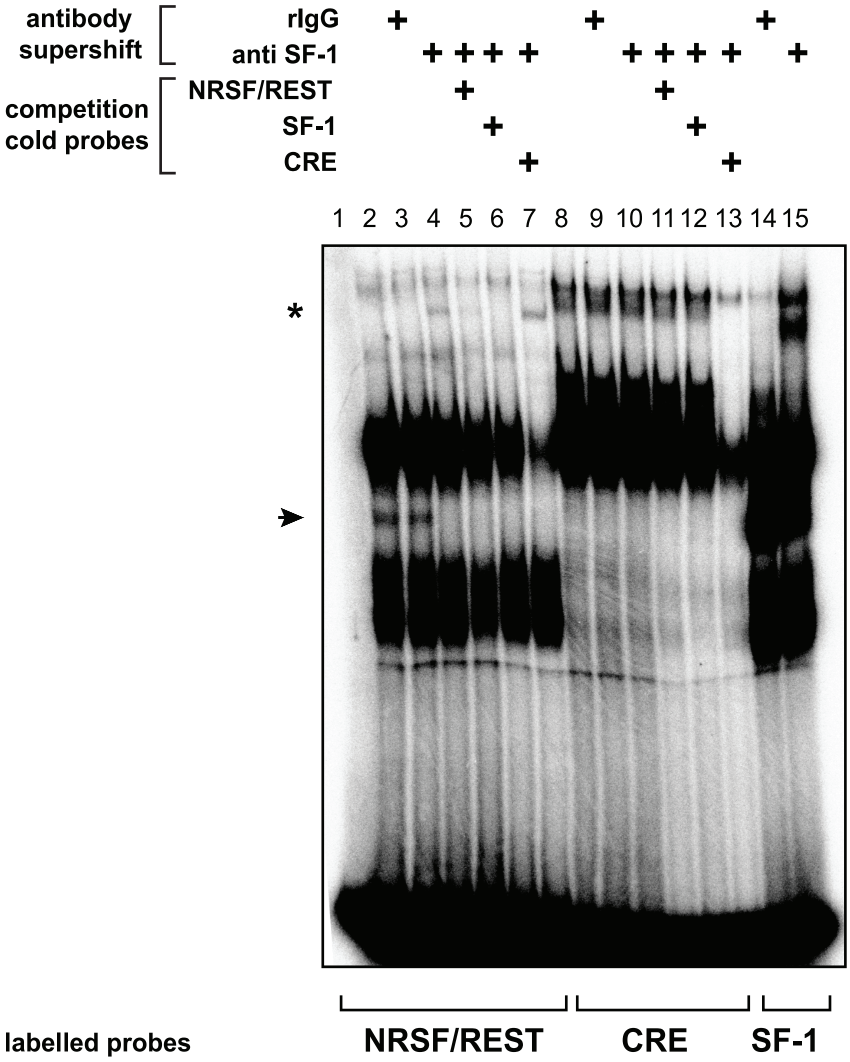

Supplement: Supplementary Data [file supp_gkt658_nar-00868-x-2013-File008.pdf]
